# Understanding the impact of a multispecialty electronic consultation service on family physician referral rates to specialists: a randomized controlled trial using health administrative data

**DOI:** 10.1186/s13063-019-3393-5

**Published:** 2019-06-10

**Authors:** Clare Liddy, Isabella Moroz, Erin Keely, Monica Taljaard, Catherine Deri Armstrong, Amir Afkham, Claire E. Kendall

**Affiliations:** 1 C.T. Lamont Primary Health Care Research Centre, Bruyère Research Institute, 43 Bruyère St. Annex E, Room 106, Ottawa, ON K1N 5C8 Canada; 2 Department of Family Medicine, University of Ottawa, Ottawa, ON Canada; 3 Department of Medicine, University of Ottawa, Ottawa, ON Canada; 4 Division of Endocrinology/Metabolism, The Ottawa Hospital, Ottawa, ON Canada; 5 Clinical Epidemiology Program, Ottawa Hospital Research Institute, Ottawa, ON Canada; 6 Department of Economics, University of Ottawa, Ottawa, ON Canada; 7 Champlain Local Health Integration Network, Ottawa, ON Canada

**Keywords:** Primary care, Specialist referral, Health systems, Wait times, Access to care

## Abstract

**Background:**

Electronic consultation (eConsult) services are secure online applications facilitating provider-to-provider communication. They have been found to improve access to specialist care. However, little is known about eConsult’s impact on family physicians’ referral rates to specialty care. The objective of this study was to assess the impact of a multispecialty eConsult service on referral rates from primary care.

**Methods:**

In this parallel-arm, randomized controlled trial, we recruited primary care providers across Ontario not previously enrolled with eConsult. We randomly assigned participants to intervention and control arms. Participants in the intervention arm received access to eConsult for a period of 1 year while those in the control arm received no access to eConsult. The main outcome was specialist referral rate, expressed as the total number of referrals to (1) specialties available through eConsult, and (2) all medical specialties, per 100 patients seen. Multivariable negative binomial regression analysis was used to evaluate the effect of the intervention before and after adjusting for provider characteristics, using health administrative data.

**Results:**

One hundred and thirteen participants were randomized (56 to control and 57 to intervention). For the primary outcome (referrals to eConsult specialties), the results show a statistically significant reduction in the number of referrals in both arms (control-arm Rate Ratio (RR), 0.85, 95% CI 0.79 to 0.91; intervention-arm RR, 0.80, 95% CI 0.74 to 0.85; unadjusted and adjusted RR values almost identical), as compared to the baseline data collected during the 12-month period before randomization, with a non-statistically significant 6% greater reduction in referrals in the intervention arm, compared to the control arm (unadjusted RR 0.94, 95% CI 0.85 to 1.03; adjusted RR 0.93, 95% CI 0.85 to 1.03).

**Conclusions:**

Our randomized controlled trial of a multispecialty eConsult service demonstrated inconclusive results in terms of the impact of eConsult on physician referral rates. Findings are discussed in light of important limitations associated with conducting randomized controlled trials (RCTs) of complex interventions in the primary care context with intent to inform the design and analysis of future trials.

**Trial registration:**

Clinicaltrials.gov, ID: NCT02053467. Registered prospectively on 3 February 2014.

## Background

Excessive wait times for specialty care are a problem facing many countries, including Canada. The 2016 Commonwealth Fund Survey reported that Canada continues to perform below the international average for timely access to patient care, with Canadians in all provinces reporting the longest wait times for specialists among the 11 countries studied [1]. More than half of Canadians (56%) waited longer than 4 weeks to see a specialist compared with the international average of 36% [1]. Excessive wait times can cause patients anxiety and stress, delay diagnosis of their condition, necessitate repeating the tests, and potentially cause further deterioration of their conditions [2], leading to higher costs and reduced satisfaction for patients and providers [2–5].

In response, innovative solutions for delivering care that are not based on face-to-face visits, such as eConsult, are gaining momentum. In 2009 our team began developing, implementing, and evaluating an innovative eHealth solution called the Champlain BASE™ (Building Access to Specialists through eConsultation) eConsult service [6]. eConsult is a form of asynchronous communication whereby primary care providers (PCP) and specialists can communicate directly about a patient through a secure web-based application. PCPs can submit a patient question (usually for a patient who would otherwise have been referred) to one of over 100 specialty services via a web-based portal. They can attach any additional information (e.g., photos, test results, Electronic Medical Record-generated letter). The case is assigned to a specialist, who receives an email notification prompting them to access the case via the secure site. Specialists are expected to provide an answer within 1 week. They can reply to the question, request additional information, or recommend a referral, and advise the PCP on other matters such as medication changes, additional tests, or other critical actions to be completed before the face-to-face specialty care appointment. PCPs ultimately decide how to apply the specialists’ suggestion and when the case can be closed. Specialists are compensated on a pro-rated hourly basis [6, 7].

The eConsult service’s effectiveness at improving access, high levels of patient and provider satisfaction, and ability to lower costs for care have been described in previous studies [6–9]. However, several knowledge gaps remain. Our recent systematic review summarizes 36 studies of eConsult services worldwide, of which half were set in the United States and 78% focused on a single specialty (commonly, dermatology) [10]. Study quality was average, with no randomized controlled trials (RCTs) conducted on multispecialty services or in a Canadian setting where access to physician services is universally funded. A systematic review conducted by Vimalananda et al. found similar results, with two thirds of all included studies emerging from the same three eConsult services [11]. Both reviews cite quick response times, avoided referrals for face-to-face visits, and high levels of provider satisfaction while noting an absence of data on clinical outcomes, cost-effectiveness and impact on referral rates for face-to-face visits [10, 11]. These findings, while promising, underscored the need for a RCT to examine the impact of a multispecialty eConsult service.

The objective of this study was to assess the impact of the Champlain BASE™ eConsult service on referral rates using an RCT of eConsult versus usual referral practices. We hypothesized that access to eConsult would lead to a decrease in referral requests for face-to-face specialist visits from PCPs in the intervention group.

## Methods

### Trial design

**Fig. 1:**
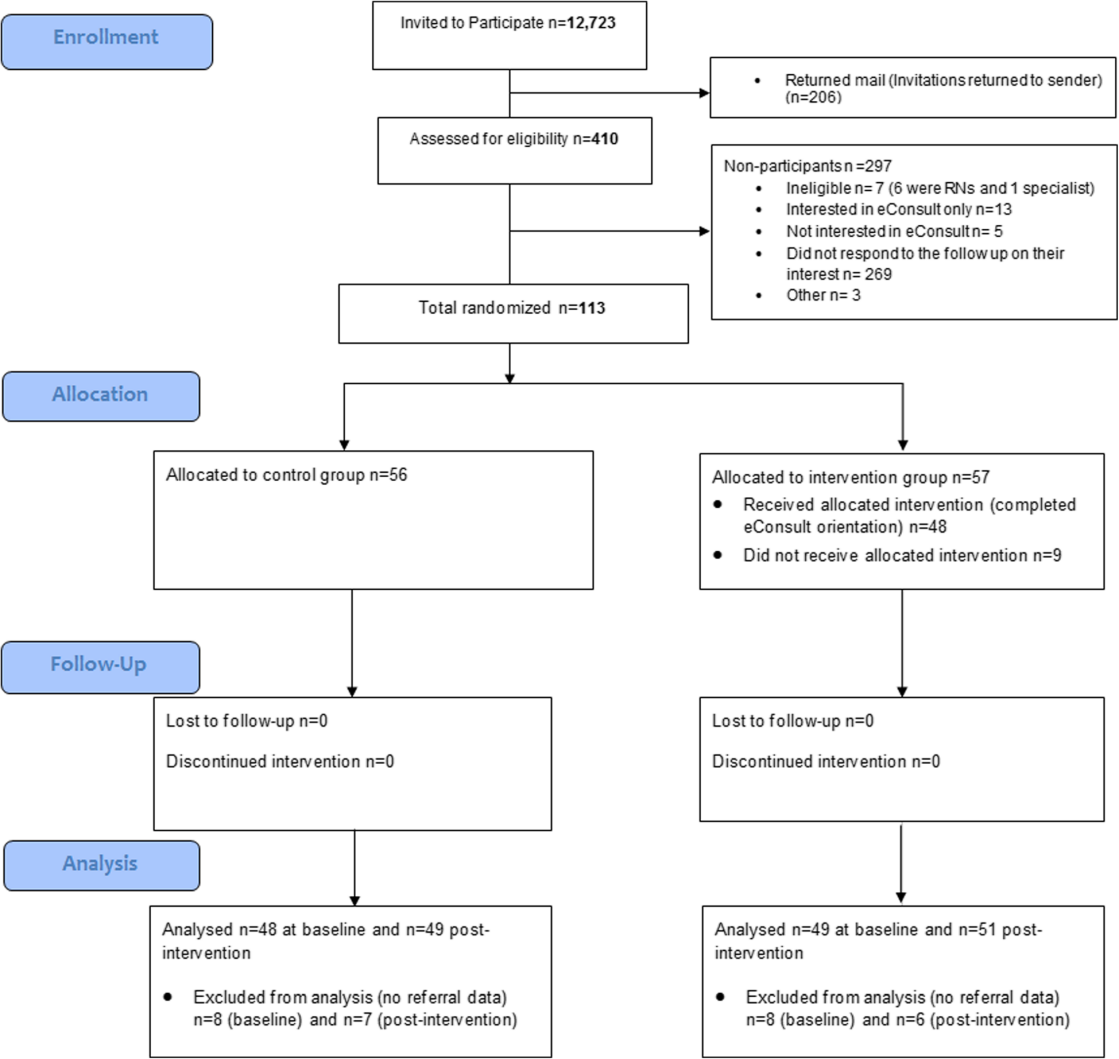
Flow of participants through trial

This was a parallel-arm RCT that recruited family physicians in Ontario to use the Champlain BASE™ eConsult service. (Fig. 1) Physicians were randomized 1:1 to either the intervention or control arm between 31 January 2014 and 26 September 2014. Although originally intended as a stepped-wedge RCT, the design was changed to a parallel-arm before and after RCT due to the fact that randomization could not be performed at a discrete time point for all participants.

### Intervention

**Fig. 2:**
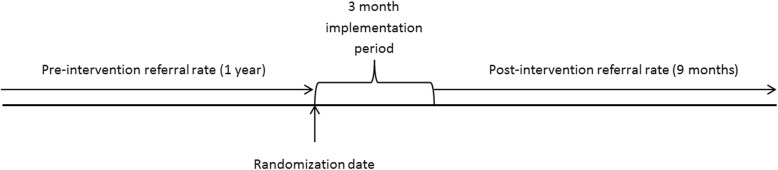
Illustration of the study design and intervention time frames

The full details of the eConsult service have been discussed elsewhere [6–8]. In brief, after undergoing registration, which includes orientation and brief training on the use of the service, users are able to submit patient-specific clinical questions to specialists through a secure, web-based application. Specialists are asked to respond within 7 days and, for each eConsult, are able to: (1) provide a recommendation, (2) request more information, or (3) recommend a face-to-face referral. The communication between PCPs and specialists is iterative and the discussion can occur back and forth until, ultimately, the PCP closes the case. Physicians randomized to the intervention group received access to eConsult right away (pending completion of an orientation session), while those randomized to the control group used standard referral practices for 1 year after randomization and then were given the option to use eConsult in the second year. The intervention period for the treatment group lasted 1 year following the enrollment/randomization period which began on 31 January 2014 and ended on 26 September (see “Randomization” section for details). As it took an average of 3 months from the randomization date for the intervention group physicians to complete the mandatory eConsult orientation and brief training, the post-randomization outcome was assessed for the following 9 months rather than for the full 12 months post randomization. The pre-randomization period was 12 months in duration for both groups. These timelines are depicted in Fig. 2.

### Participants

All Ontario family physicians who were not already registered for the eConsult service were eligible to participate.

### Setting

This study was conducted in Ontario, Canada, the country’s most populous province, with publicly funded, universal access to physician services to its over 13 million residents [12].

### Recruitment

Every eligible family physician in the province of Ontario (not already using eConsult service) was sent a two-page information brochure outlining the eConsult service, along with a letter describing the research study between January 2014 and April 2014. Recruitment was conducted by a third party (Ontario Medical Association); the eConsult research team did not have access to the mail-out list. Family physicians interested in participating in the study were asked to contact the study team for more information. Those expressing interest were sent recruitment packages which included a study information sheet, a consent form, and a practice survey. Three weeks later, a follow-up letter and recruitment package was sent again by fax/email to the non-responders. Recruitment continued until the target sample (minimum *n* = 50 physicians/group) was reached.

### Randomization

Individual physicians were the units of randomization. A computer-generated random list of numbers was created using an online website (https://www.randomizer.org/) to allocate participants to either the control or the intervention arm. The allocation sequence was implemented by a research staff member not involved in this research project and concealed from the researchers and staff responsible for enrolling and assessing participants in sequentially numbered, opaque, sealed, and stapled envelopes. Due to the nature of the intervention, participating physicians were not blinded to their allocation status. No stratification was used since we recruited individual physicians (rather than practices) into the trial.

### Outcomes

Our primary outcome was specialist referral rate defined as the total number of referrals for face-to-face visits to all medical specialties available through eConsult service during the study period (see Appendix) per 100 unique patients (not encounters) seen. The denominator “patients seen” included patients who were seen at least once during the assessment period (baseline or post intervention). The secondary outcome was referral rate to all medical specialties. Only referrals for face-to-face specialist visits initiated by the physicians in the trial were counted. If a patient was referred to the same specialist multiple times during our study period, this was counted as a single referral. If the patient was referred to two physicians of the same specialty in our time period, this was counted as two referrals. Referrals to other family physicians were excluded.

### Sample size calculation

A total sample size of 80,000 patients seen (50 providers in each arm with an average of 800 patients per provider), was calculated to achieve 80% power to detect an absolute reduction in referrals of 6 per 100 patients from a control-arm referral rate of 31 per 100 patients, estimated from a previous study [13], using a two-sided significance level of 5% and assuming a between-provider coefficient of variation of 0.38 [14].

### Data sources

We used the following databases from the Institute for Clinical Evaluative Sciences (ICES, ices.on.ca) to obtain physician and patient characteristics: Registered Persons Database, containing demographic data for all residents eligible for provincial health care; Ontario Health Insurance Program (OHIP) billing claims system capturing approximately 95% of physician services in Ontario; the Client Agency Program Enrollment Registry and Corporate Provider Database, for patient enrollment with individual primary care physicians; and the ICES Physician Database, containing physician demographic information, training, and practice setting. 2006 Statistics Canada Census data was used to assign income quintile to patients based on their postal code. These data sets were linked using unique encoded identifiers and analyzed at ICES.

We also used the Champlain BASE™ eConsult utilization data, which was routinely collected throughout the study period. The data was used to identify the physicians in each group (treatment and control) as well as the date of randomization which was used to identify the relevant 12-month pre-intervention period and the 9-month post-intervention period, as per Fig. 2. Randomization took place between 31 January 2014 and 26 September 2014. The data was transferred to ICES and linked to the administrative data using the encrypted College of Physicians and Surgeons of Ontario license number for each physician. All analyses were carried out at ICES. The study was registered at Clinicaltrials.gov (NCT02053467).

### Creation of patient rosters

We used the following process to construct patient rosters for each physician in the study. Patients officially rostered to each physician in the trial were identified using the Client Agency Program Enrollment Database. For patients who were not rostered to a physician, they were “virtually” rostered through the standard approach based on the physician who had billed the largest dollar amount over a 2-year period. Both physician and practice characteristics were obtained from the ICES Physician Database. Patient characteristics, including age, sex, and residential postal code, were obtained from the Registered Persons Database. Additionally, census data was used to assign patients to both income quintile (with lowest = 1, highest = 5) and rurality category based on the Rurality Index of Ontario scores, and categorized as urban (0–10), non-urban (10–39), and rural (40+). We assigned comorbidity using the Johns Hopkins Adjusted Clinical Groups Case-Mix Assignment software (The Johns Hopkins ACG® System Version 10) by assigning from 0 up to 32 distinct Aggregated Diagnosis Groups (ADGs), with a higher number of ADGs reflecting a higher level of diagnosed comorbidity [15].

### Data analysis

All analyses estimate intention to treat (ITT) effects. Physicians were excluded from the analysis only if (1) they had no OHIP billings during the study period, and/or (2) they had less than 100 patients rostered to them on the randomization date. Both cases preclude examination of their referral rates due to lack of data.

Descriptive statistics were generated to describe patient and physician characteristics at the time of randomization for the two groups: eConsult and control. Provider characteristics were described using frequency and percent for categorical variables, or mean and standard deviation for continuous variables. Patient characteristics were aggregated to the level of the provider and described using mean and standard deviation.

The primary outcome, referral rate per 100 patients seen, was analyzed using a repeated measures multivariable random effects negative binomial regression model. The unit of analysis was the provider. The log link function was used and the natural log of the number of patients seen in each study period was included as an offset term. Intervention, study period, and the interaction between intervention and study period were specified as fixed covariates. The correlation in repeated measures on the same physician was accounted for by specifying a compound symmetric covariance matrix. The nesting of multiple providers in the same practice was additionally accounted for by specifying a random intercept for the practice. To account for underlying secular/time trends in the rate of referrals over the study period, the effect of the intervention was expressed as the between-arm difference in the change in referral rates from pre to post intervention. By exponentiating the regression coefficient for the eConsult by study year interaction term, we obtained this estimate as a Rate Ratio (RR) together with 95% confidence interval (CI). Least square mean estimates of the model-based referral rates in each arm in the pre- and post-intervention periods were obtained from the models. The models were adjusted for physician characteristics defined after inspecting baseline differences between the groups, but before implementing the primary outcome analysis: years since graduation, location of medical training, and practice model (Capitation: Interdisciplinary (Family Health Team (FHT))/Capitation: Non-interdisciplinary (not FHT)/Reformed Fee for service, Traditional Fee for service, other).

Because it is known that an analysis of change from baseline can be overly sensitive to baseline differences between the arms [16], we also conducted a secondary sensitivity analysis of covariance (ANCOVA) of the primary outcome post intervention by specifying the log of the provider’s baseline referral rate as a covariate. Results of these analyses were expressed as Rate Ratios (intervention versus control-arm referral rates), adjusted for the baseline referral rate.

SAS Version 9.4 (SAS Institute, Cary, NC, USA) was used for all analyses.

## Results

All eligible family physicians (12,723) in Ontario were invited, of whom 113 consented to participate in the eConsult trial and were randomized to the intervention (*n* = 57) and control groups (*n* = 56). However, gaps in administrative data caused some physician to be partially or fully excluded, and consequently among intervention group physicians only 49 physicians were analyzed pre intervention and 51 post intervention; in the control group, 48 physicians were analyzed pre intervention and 49 post intervention. The flow of participants through the trial is presented in Fig. 1.

Table 1 presents provider and patient characteristics for the intervention and control groups at the time of randomization. Patient panels were comparable between the groups. At the provider level, it can be seen that a higher proportion of intervention (eConsult) group physicians were practicing in the Interdisciplinary (FHT) model and in group practices with two to five physicians. eConsult physicians had a median of 20 years since graduation compared to control with 23 years.

**Table 1:** Participant characteristics

Characteristics	Control *N* = 49	eConsult *N* = 52
Provider level		
Female (*n*, %)	31 (63.3)	36 (69.2)
Age at randomization (mean, SD)	48.45 (11.12)	46.67 (10.08)
Years since graduation (mean, SD)	22.9 (11.71)	19.83 (10.37)
Foreign trained (*n*, %)	9 (18.4)	6 (11.5)
Model type (*n*, %)		
Capitation^a^: Interdisciplinary (Family Health Team (FHT))	20 (40.8%)	14 (26.9%)
Capitation: Non-interdisciplinary (not FHT)	20 (40.8%)	27 (51.9%)
Reformed Fee for service)	7 (14.3%)	10 (19.2%)
Traditional Fee for service	≤ 5 (2.0%)	≤ 5 (1.9%)
Other	≤ 5 (2.0%)	0 (0.0%)
Rural (*n*, %)	14 (28.6%)	14 (26.9%)
Number of physicians in group practice (*n*, %)		
1	24 (49%)	19 (36.5%)
2–5	12 (24.5%)	22 (42.3%)
> 5	11 (22.4%)	9 (17.3%)
missing	≤ 5 (4.1%)	≤ 5 (3.8%)
Number of patients in roster	63,905	59,241
Patient level, aggregated to physician level		
Proportion female (mean, SD)	0.55 (0.11)	0.56 (0.10)
Proportion age category (years: mean, SD)		
0–17	0.19 (0.08)	0.21 (0.13)
18–25	0.10 (0.02)	0.10 (0.03)
26–40	0.18 (0.07)	0.19 (0.07)
41–65	0.36 (0.06)	0.37 (0.06)
66+	0.16 (0.07)	0.15 (0.07)
Proportion in income quintile (mean, SD)		
1	0.13 (0.08)	0.15 (0.12)
2	0.18 (0.06)	0.15 (0.06)
3	0.21 (0.07)	0.18 (0.07)
4	0.22 (0.08)	0.22 (0.07)
5	0.27 (0.12)	0.30 (0.17)
Proportion rural (mean, SD)	0.28 (0.37)	0.24 (0.35)
Mean number of ADGs in the 2 years prior to randomization (mean, SD)	5.90 (0.61)	6.03 (0.70)
Mean number of primary care visits in year prior to cohort entry (mean, SD)	4.52 (1.71)	4.25 (1.14)

ADGs: Aggregated Diagnosis Groups

^a^Capitation is a payment model in which providers are remunerated for the number of patients enrolled in their care, regardless of whether or not the patient sees them in a given period

Participants completed 44,066 referrals across all medical specialties during the study period: 22,079 by the eConsult physicians and 21,987 by the control physicians. The eConsult physicians and control physicians referred most frequently to the same five specialties before and after intervention: dermatology, general surgery, obstetrics and gynecology, gastroenterology, otolaryngology (see Table 2 for detailed breakdown).

**Table 2:** Distribution of specialist referrals (overall and by eConsult exposure)

Specialty group	Pre intervention		Post intervention		Overall
	Control % (*n*)	eConsult % (*n*)	Control % (*n*)	eConsult % (*n*)	% (*n*)
Dermatology	10.1 (1276)^1^	11.3 (1447)^1^	8.7 (809)^3^	9.7 (894)^2^	10.0 (4426)^1^
General surgery	9.0 (1145)^2^	9.6 (1228)^3^	10.1 (937)^1^	10.0 (921)^1^	9.6 (4231)^1^
Obstetrics and gynecology	8.9 (1135)^3^	10.1 (1296)^2^	9.0 (833)^2^	9.4 (872)^3^	9.4 (4136)^3^
Gastroenterology	8.6 (1088)^4^	7.2 (922)^5^	8.3 (767)^4^	7.0 (649)^4^	7.8 (3426)^4^
Otolaryngology	6.7 (853)^5^	7.4 (945)^4^	6.9 (645)^5^	6.3 (581)^5^	6.9 (3024)^5^
Orthopedic surgery	6.2 (787)	6.1 (787)	5.9 (544)	5.5 (513)	6.0 (2631)
Cardiology	5.0 (636)	4.9 (635)	4.6 (423)	5.2 (481)	4.9 (2175)
Internal medicine	4.9 (623)	4.6 (587)	5.1 (476)	5.1 (468)	4.9 (2154)
Urology	4.3 (552)	4.6 (595)	3.9 (366)	4.3 (397)	4.3 (1910)
Neurology	4.3 (542)	3.7 (481)	4.4 (407)	4.0 (367)	4.1 (1797)
Psychiatry	3.2 (410)	3.5 (453)	2.6 (239)	3.6 (331)	3.3 (1433)
Respirology	3.3 (415)	3.0 (384)	3.0 (281)	3.2 (295)	3.1 (1375)
Plastic surgery	3.4 (428)	2.7 (351)	3.2 (301)	2.6 (243)	3.0 (1323)
Ophthalmology	2.7 (349)	2.3 (289)	3.1 (284)	2.0 (185)	2.5 (1107)
Pediatrics	2.8 (355)	2.0 (253)	2.2 (209)	2.2 (205)	2.3 (1022)
Physical medicine and rehab	2.0 (258)	2.5 (322)	2.2 (206)	2.4 (218)	2.3 (1004)
Rheumatology	2.2 (278)	2.3 (290)	2.0 (188)	1.7 (158)	2.1 (914)
Endocrinology	1.8 (231)	1.6 (205)	1.9 (172)	1.8 (163)	1.7 (771)
Clinical immunology	1.6 (207)	2.3 (299)	1.1 (102)	1.7 (158)	1.7 (766)
Unknown	0.1 (19)	0.1 (14)	3.8 (352)	4.1 (379)	1.7 (764)
Hematology	1.3 (162)	1.4 (186)	1.2 (108)	1.2 (112)	1.3 (568)
Pediatric clinical immunology	0.9 (120)	1.1 (135)	1.0 (96)	1.2 (114)	1.1 (465)
Nephrology	0.9 (114)	0.8 (109)	0.8 (74)	0.8 (76)	0.8 (373)
Vascular surgery	0.9 (108)	0.9 (111)	0.8 (77)	0.6 (58)	0.8 (354)
Anesthesiology	0.9 (116)	0.5 (64)	0.6 (60)	0.7 (68)	0.7 (308)
Geriatric medicine	0.6 (74)	0.6 (75)	0.5 (44)	0.6 (54)	0.6 (247)
Neurosurgery	0.5 (62)	0.4 (50)	0.6 (57)	0.5 (47)	0.5 (216)
Diagnostic radiology	0.5 (62)	0.4 (48)	0.5 (46)	0.4 (35)	0.4 (191)
Infectious diseases	0.5 (65)	0.4 (45)	0.5 (48)	0.3 (31)	0.4 (189)
Thoracic surgery	0.3 (35)	0.4 (47)	0.3 (29)	0.4 (34)	0.3 (145)

Notes

Only specialties with more than 100 referrals overall are shown

The superscript numbers indicate the rank order of number of referrals

Table 3 presents the observed mean and standard deviation of numbers of patients seen, as well as numbers of referrals to eConsult specialties and to all specialties during the baseline and post-randomization assessment periods. It also presents the observed referral rates per 100 patients seen for eConsult and for all specialties including those not available via eConsult (see “Appendix”). There were differences between the groups pre intervention: the eConsult group had slightly lower mean number of total referrals than the control group for the specialties available via eConsult (244.00, standard deviation (SD) = 142.88 versus 246.10, SD = 161.97) and all specialties (261.86, SD = 151.88 versus 264.48, SD = 172.95 respectively), but higher mean number of referrals per 100 patients seen for specialties available via eConsult (32.83, SD = 12.40 versus 28.15, SD = 10.89) and all specialties (35.24, SD = 13.20 versus 30.20, SD = 11.60). Post intervention, both groups showed decreases in their mean number of referrals and mean referral rates per 100 patients.

**Table 3:** Observed mean numbers of referrals, numbers of patients seen, and referral rates per 100 patients seen, for all specialties and for eConsult specialties in the pre- and post-intervention periods and by study arm

	Control		eConsult	
	Pre (*n* = 48)	Post (*n* = 49)	Pre (*n* = 49)	Post (*n* = 51)
Number of patients seen mean, (SD), range	828 (425) 93 to 1971	724 (370) 11 to 1692	716 (278) 114 to 1237	608 (258) 90 to 1134
Number of referrals mean, (SD)				
eConsult specialties	246 (162)	169 (93)	244 (143)	162 (99)
All specialties	264 (173)	190 (103)	262 (152)	181 (109)
Referral rates (per 100 patients seen)				
eConsult specialties	28.15 (10.89)	23.42 (7.76)	32.83 (12.40)	25.83 (9.97)
All specialties	30.19 (11.59)	26.18 (8.59)	35.23 (13.21)	29.02 (11.04)

Table 4 shows the multivariable negative binomial regression analyses of the primary and secondary outcomes. Between-arm differences in the pre- and post-intervention periods, as well as differences in change from the pre-intervention arm, are presented as unadjusted and adjusted RRs together with 95% confidence intervals (CI). For the primary outcome (referrals to eConsult specialties), the results show a non-statistically significant 6% greater reduction in referrals in the intervention arm, relative to the control arm (unadjusted RR 0.94, 95% CI 0.85 to 1.03) and a non-statistically significant 7% relative reduction after adjusting for covariates (adjusted RR 0.93, 95% CI 0.85 to 1.03). The differences between the arms were statistically significant only at baseline (*p* = 0.03). While there was a statistically significant decrease in referral rates from pre to post intervention in the control arm (unadjusted and adjusted RRs 0.85, 95% CIs 0.79 to 0.91) there was also a decrease in referral rates in the intervention arm (unadjusted RR 0.80, adjusted RR 0.79, 95% CIs 0.79 to 0.91). As the change in referral behavior over time in the control arm cannot be the result of eConsult, this suggests an underlying decreasing secular trend in referrals.

**Table 4:** Unadjusted and adjusted^a^ multivariable regression analyses of primary and secondary outcomes

	Unadjusted			^a^Adjusted		
	RR	95% CIs	*p* value	RR	95% CIs	*p* value
Referrals to eConsult specialties (primary outcome)						
Between-arm (control vs eConsult) difference, pre intervention	0.88	0.79–0.99	0.03	0.89	0.79–0.99	0.03
Between-arm difference, post intervention	0.94	0.84–1.06	0.30	0.95	0.85–1.06	0.34
Control arm, change from pre to post intervention	0.85	0.79–0.91	< 0.0001	0.85	0.79–0.91	< 0.0001
Intervention arm, change from pre to post intervention	0.80	0.74–0.85	< 0.0001	0.79	0.74–0.85	< 0.0001
Between-arm differences in pre- to post-intervention change	0.94	0.85–1.03	0.18	0.93	0.85–1.03	0.17
Overall referrals (secondary outcome)						
Between-arm difference, pre intervention	0.88	0.79–0.99	0.03	0.89	0.79–0.99	0.03
Between-arm difference, post intervention	0.94	0.84–1.05	0.25	0.94	0.84–1.05	0.30
Control arm, change from pre to postintervention	0.89	0.83–0.95	0.0006	0.89	0.82–0.95	0.001
Intervention arm, change from pre to post intervention	0.83	0.78–0.89	< 0.0001	0.83	0.78–0.89	< 0.0001
Between-arm differences in pre- to post-intervention change	0.94	0.85–1.04	0.22	0.94	0.85–1.04	0.22

Comparator = control arm, RR < 1 means a lower referral rate (or change in referral rates) in the intervention arm than in the control arm

^a^All regression models were adjusted for the following physician characteristics: years since graduation, location of medical training, and practice model (Capitation: Interdisciplinary (Family Health Team (FHT))/Capitation: Non-interdisciplinary (not FHT)/Reformed Fee for service, Traditional Fee for service, other)

Note: List of specialties available on eConsult at the beginning of the study available in “Appendix”

CI confidence interval, RR Rate Ratio

Results were similar for the secondary outcome (referrals to all specialties). Overall, adjusting for provider characteristics did not make much difference in the rate ratios, and 95% confidence intervals barely crossed the value of 1.

Model-based unadjusted and adjusted referral rates, together with 95% CIs, are presented in Table 5. Referrals to specialties available via eConsult decreased in both arms: from 0.31 (95% CI 0.28–0.35) to 0.25 (95% CI 0.22–0.27) in the intervention arm versus 0.27 (95% CI 0.25–0.30) to 0.23 (95% CI 0.21–0.26) in the control arm, unadjusted. Adjusting for provider characteristics did not affect this finding, and similar referral rates patterns were obtained for referrals to all specialties (not restricted to those available via eConsult).

**Table 5:** Model-based unadjusted and adjusted referral rates per 100 patients seen in each study arm during the pre- and post-intervention periods with 95% confidence interval

	Control		eConsult	
	Pre (*n* = 48)	Post (*n* = 49)	Pre (*n* = 49)	Post (*n* = 51)
Referral rates to eConsult specialties				
Unadjusted	0.27 (0.25–0.30)	0.23 (0.21–0.26)	0.31 (0.28–0.35)	0.25 (0.22–0.27)
Adjusted	0.28 (0.25–0.30)	0.23 (0.21–0.26)	0.31 (0.28–0.34)	0.25 (0.22–0.27)
Referral rates to all specialties				
Unadjusted	0.30 (0.27–0.33)	0.26 (0.24–0.29)	0.34 (0.31–0.37)	0.28 (0.25–0.31)
Adjusted	0.30 (0.27–0.33)	0.26 (0.24–0.29)	0.33 (0.30–0.37)	0.28 (0.25–0.31)

The results from the secondary ANCOVA analysis for the primary (referrals to eConsult specialties) and secondary (referrals to all specialties) outcomes are presented in Table 6 as Rate Ratios (RR) together with 95% CIs and show no statistically significant difference between the groups. In the “unadjusted,” ANCOVA analysis, only the provider’s pre-intervention referral rates were used as a covariate, while in the “adjusted,” version, all the remaining baseline covariates were included as well.

**Table 6:** Unadjusted and adjusted^a^ analysis of covariance (ANCOVA) analyses of primary and secondary outcomes

ANCOVA estimates	Unadjusted		Adjusted^a^	
	RR	95% CIs	RR	95% CIs
Referrals to eConsult Specialties (primary outcome)	1.00	0.91–1.11	0.99	0.89–1.10
Overall Referrals (secondary outcome)	1.01	0.91–1.12	0.99	0.89–1.10

^a^Rate Ratios (RR) (intervention versus control-arm referral rates) adjusted for the provider’s pre-intervention referral rate in addition to all the remaining covariates (years since graduation, location of medical training, and practice model (Capitation: Interdisciplinary (Family Health Team (FHT)/Capitation: Non-interdisciplinary (not FHT)/Reformed Fee for service, Traditional Fee for service, other)

*CIs* confidence intervals

## Discussion

This RCT examined the effect of using a multispecialty eConsult service on referrals from primary to specialty care. We hypothesized that eConsult would lead to a decrease in the overall number of referrals in the intervention arm. Our analysis showed a statistically significant reduction in the number of referrals in both arms, with a non-significant 6% greater reduction in the eConsult intervention arm compared to the control arm after accounting for provider characteristics when analyzed using a difference in difference methodology. Given the significant imbalance in referral rates between the groups observed at baseline, a secondary sensitivity analysis using ANCOVA was conducted and revealed that the post-intervention difference between the groups after accounting for pre-intervention values was not statistically significant.

As we reflect on the implementation of the trial, there are several limitations and challenges associated with conducting an RCT of a complex intervention such as eConsult in a primary care context that have to be acknowledged. RCTs may minimize rather than eliminate bias [17]. The participants in our study had to manifest their interest to be considered for enrollment and our final sample represented less than 1% of the total population invited to participate in the trial. Our groups were significantly imbalanced at baseline despite randomization, introducing a chance bias into the study. This is significant given the tremendous documented variability in physician referral rates and the methodological challenges of studying referral patterns in primary care, discussed in our previous paper [18]. As discussed in depth by Fives et al. (2013), random allocation does not ensure baseline equality [19]. Its role is to protect against selection bias, as allocation is not based on any systematically biased method. However, as was the case in our study, a statistically significant baseline differences can still occur. One way to deal with baseline imbalance would be to attempt to reduce the likelihood of it occurring by modifying the random allocation process by using “stratified” or “blocked” randomization. We recommend doing so in the future studies, though it has been argued that allocation of participants in a stratified study is still random, hence does not guarantee equivalence on relevant variables. RCTs often suffer from two major complications: non-compliance and missing outcomes, to which ITT analysis, which we employed, offers a potential solution [20]. With respect to non-compliance, even though in an ideal scenario every participant enrolled in RCT would complete their allocated treatment, nine out of 57 physicians (16%) in the eConsult intervention arm did not complete the eConsult orientation and hence were not exposed to the intervention. Furthermore, the eConsult group completed a total of 413 eConsults (or 8.6 per each “exposed” physician) by the end of the study period (data not shown). This begs two questions. First, did our intervention group receive enough of a “dose” of the eConsult to impact their referral rates? Second, was our study time frame too short to detect the impact of the eConsult intervention? The answers to both questions are unknown as these ideas have not yet been a topic of empirical study. With respect to missing outcomes, due to limitations related to the use of administrative health databases (e.g., unavailability of health administrative data for certain physicians in certain study years), we did not end up with 50 providers per arm resulting in an underpowered trial. This may have been affected by the fact that administrative data: (1) are not complete since only 95% of outpatient physician encounters are recorded [21]; (2) contain no referral data for salaried primary care physicians working in community health centres; and (3) rely on imperfect algorithms to create patient rosters.

While the results of our study did not identify a statistically significant impact on PCP referral patterns, a growing body of international literature on telemedicine systems from various countries including United States (eConsult and eReferral), Brazil (Telehealth Network of Minas Gerais), and the international non-governmental organization Médecins Sans Frontières show that 19 to 68% of eConsult requests are resolved without the need for an in-person specialty visit [10, 11, 22]. Given these findings, it is reasonable to assume that frequent use of eConsult has the potential to lead to a lower overall number of referrals. Afterall, even though non-statistically significant, a 6% greater reduction in referrals in the intervention arm, relative to the control arm, can be clinically significant, especially on a population level, and is worth mentioning in the context of health services delivery improvement efforts. This potential effect on reducing face-to-face visits can be attributed, in part, to the fact that the rapid access to specialist expertise afforded through eConsult supports the patient and the PCP in not only continuing management of their current patient (thus supporting a patient medical home approach and improved continuity of care), but also by offering educational value through case-based learning, thus increasing the provider’s repertoire of expertise potentially impacting the need to refer for future patients [23–25]. Unfortunately, the effects of eConsult on primary care physician referral behavior have not been previously studied utilizing rigorous methodology. There have been only a few randomized trials of eConsult limited to a single specialty and none assessed impact on overall referral rates [26–28]. To our knowledge, the present trial is unique in that it is the first RCT examining the effect of a multispecialty eConsult model on physician behavior, with the main outcome of referral rates. Unfortunately, our results were inconclusive and given a small sample size, a possibility of type 2 error (or a false negative finding) must be acknowledged. Future trials examining this impact of eConsult on referral rate should be conducted and include a larger sample size to protect against selection bias, stratification at the level of the organization (e.g., primary care clinic), employing specific strategies aimed to optimize implementation of the intervention (e.g., a run-in period where those in the intervention group commit to submitting at least one eConsult to maximize compliance) [29], and finally a longer trial period. In addition, it would be interesting to examine if the use of eConsult services shortens the time to see specialists when a face-to-face referral is recommended from the eConsult communications.

eConsult remains an innovative solution that has the potential to transform the primary–specialty care interface by enabling a move away from reliance on face-to-face specialty visit-based care, addressing the documented fragmentation and care-coordination challenges, reducing the number of specialty care visits for conditions that can be managed by PCPs, increasing the effectiveness of face-to-face specialty visits when they occur, and producing cost savings [10, 11]. It is a complex intervention to study in a rigorous manner, and the evidence shows that many complex interventions that show promising effects in smaller studies (usually observational and uncontrolled) are not replicated in RCTs [30]. While our results were not statistically significant, our trial provides important methodological insight to guide others seeking to implement and evaluate electronic consultation services.

## Conclusion

This is the first RCT of a multispecialty eConsult service which aimed to demonstrate that the use of eConsult may be associated with fewer referrals from primary to specialist care. Due to various challenges associated with conducting a randomized controlled trial of a complex intervention in the primary care context, our conclusions are limited. Nonetheless, the results and reflections on the limitations encountered should prove invaluable in informing the planning of future trials to examine the effects of eConsult on physician referral behavior and the resulting population-level impacts. Further research is prudent to demonstrate that any reductions in the rate of referral are appropriate and of comparable quality to in-person consultations as well as to examine cost effectiveness to our single-payer system.

## Data Availability

The data set from this study is held securely in coded form at the Institute for Clinical Evaluative Sciences (ICES). While data-sharing agreements prohibit ICES from making the data set publicly available, access may be granted to those who meet pre-specified criteria for confidential access, available at www.ices.on.ca/DAS.

## Appendix

### List of eConsult specialties available at the beginning of the study period

1. Anesthesiology
2. Cardiology
3. Dermatology
4. Endocrinology
  - Paediatric Endocrinology
5. Otolaryngology
6. Gastroenterology
  b. Paediatric Gastroenterology
7. Medical Genetics
8. Haematology
9. Infectious Diseases
  - Clinical Immunology
  - Paediatric Clinical Immunology
  - Paediatric Infectious Diseases
10. Internal Medicine
11. Nephrology
  - Paediatric Nephrology
12. Neurology
13. Obstetrics and Gynaecology
14. Orthopaedic Surgery
15. Psychiatry
16. Respirology
17. Rheumatology
  - Paediatric Rheumatology
18. Urology
19. Paediatric Cardiology
20. Paediatrics
21. Paediatric Haematology/Oncology
22. Paediatric Neurology
23. Paediatric Respirology
24. Diagnostic Radiology

## Abbreviations

ADG: Aggregated Diagnosis Group
BASE™: Building – Access to Specialists through eConsultation
FHT: Family Health Team
ICES: Institute of Clinical Evaluative Sciences
ITT: Intention to treat
OHIP: Ontario Health Insurance Program
PCP: Primary care provider
RCT: Randomized Controlled Trial

## References

[1] Canadian Institute for Health Information (2017). How Canada compares: results from the Commonwealth Fund’s 2016 international health policy survey of adults in 11 countries.

[2] Canadian Intitute for Health Information (2012). Health care in Canada, 2012: a focus on wait times.

[3] Barua B, Esmail N (2013). Waiting your turn: wait times for health care in Canada.

[4] Chen AH, Yee HF (2009). Improving the primary care to specialty care interface: getting from here to there. Arch Intern Med.

[5] Barua B, Esmail N, Jackson T (2014). The effect of wait times on mortality in Canada.

[6] Liddy C, Rowan MS, Afkham A, Maranger J, Keely E (2013). Building access to specialist care through e-consultation. Open Med.

[7] Liddy C, Maranger J, Afkham A, Keely E (2013). Ten steps to establishing an e-consultation service to improve access to specialist care. Telemed J E Health.

[8] Keely E, Liddy C, Afkham A (2013). Utilization, benefits, and impact of an e-consultation service across diverse specialties and primary care providers. Telemed J E Health.

[9] Liddy C, Drosinis P, Deri Armstrong C, McKellips F, Afkham A, Keely E (2016). What are the cost savings associated with providing access to specialist care through the Champlain BASE eConsult service? A costing evaluation. BMJ Open.

[10] Liddy C, Drosinis P, Keely E (2016). Electronic consultation systems: worldwide prevalence and their impact on patient care—a systematic review. Fam Pract.

[11] Vimalananda VG, Gupte G, Seraj SM, Orlander J, Berlowitz D, Fincke BG (2015). Electronic consultations (e-consults) to improve access to specialty care: a systematic review and narrative synthesis. J Telemed Telecare.

[12] Statistics Canada. Population by year, by province and territory, CANSIM, table 051–001. http://www.statcan.gc.ca/tables-tableaux/sum-som/l01/cst01/demo02a-eng.htm. Accessed 30 Apr 2018.

[13] Alexander G (2011). eReferral strategy white paper: clearing the communications fog.

[14] Franks P, Williams GC, Zwanziger J, Mooney C, Sorbero M (2000). Why do physicians vary so widely in their referral rates?. J Gen Intern Med.

[15] Johns Hopkins University. The Johns Hopkins ACG System. https://www.johnshopkinssolutions.com/solution/acgsystem/. Accessed 30 Apr 2018.

[16] Hooper R, Forbes A, Hemming K, Takeda A, Beresford L (2018). Analysis of cluster randomised trials with an assessment of outcome at baseline. BMJ.

[17] Kaptchuk TJ (2001). The double-blind, randomized, placebo-controlled trial: gold standard or golden calf?. J Clin Epidemiol.

[18] Liddy C, Moroz I, Keely E, Taljaard M, Mark Fraser A, Deri Armstrong C (2017). The use of eConsult is associated with lower specialist referral rates: a cross sectional study using population-based health administrative data. Fam Pract.

[19] Fives A (2013). The role of random allocation in randomized controlled trials: distinguishing selection bias from baseline imbalance. J MultiDiscip Eval.

[20] Gupta SK (2011). Intention-to-treat concept: a review. Perspect Clin Res.

[21] Chan BT, Schultz SE (2005). Supply and utilization of general practitioner and family physician services in Ontario.

[22] Soriano Marcolino M, Minelli Figueira R, Pereira Afonso dos Santos J, Silva Cardoso C, Luiz Ribeiro A, Alkmim MB (2016). The experience of a sustainable large scale Brazilian telehealth network. Telemed J E Health.

[23] Liddy C, Afkham A, Drosinis P, Joschko J, Keely E (2015). Impact and satisfaction with a new eConsult service: a mixed methods study of primary care providers. J Am Board Fam Med.

[24] Barnett ML, Yee HF, Mehrotra A, Giboney P (2017). Los Angeles Safety-Net Program eConsult system was rapidly adopted and decreased wait times to see specialists. Health Aff.

[25] Kirsh SR, Ho PM, Aron DC (2014). Providing specialty consultant expertise to primary care: an expanding spectrum of modalities. Mayo Clin Proc.

[26] Whited JD, Hall RP, Foy ME, Marbrey LE, Grambow SC, Dudley TK (2004). Patient and clinician satisfaction with a store-and-forward teledermatology consult system. Telemed J E Health.

[27] Olayiwola JN, Anderson D, Jepeal N, Aseltine R, Pickett C, Yan J (2016). Electronic consultations to improve the primary care-specialty care interface for cardiology in the medically underserved: a cluster-randomized controlled trial. Ann Fam Med.

[28] Golberstein E, Kolvenbach S, Carruthers H, Druss B, Goering P. Effects of electronic psychiatric consultations on primary care provider perceptions of mental health care: survey results from a randomized evaluation. Healthcare. 2017; [In press]. 10.1016/j.hjdsi.2017.01.002 28162990

[29] Levati S, Campbell P, Frost R, Dougall N, Wells M, Donaldson C (2016). Optimisation of complex health interventions prior to a randomised controlled trial: a scoping review of strategies used. Pilot Feasibility Stud.

[30] Campbell NC, Murray E, Darbyshire J, Emery J, Farmer A, Griffiths F (2007). Designing and evaluating complex interventions to improve health care. BMJ.

